# Long-term outcome and quality of life of patients treated in surgical intensive care: a comparison between sepsis and trauma

**DOI:** 10.1186/cc5047

**Published:** 2006-09-16

**Authors:** Helena Korošec Jagodič, Klemen Jagodič, Matej Podbregar

**Affiliations:** 1 Department of Anesthesiology and Intensive Therapy, General Hospital Celje, Oblakova ulica, 3000 Celje, Slovenia; 2 Department of Urology, General Hospital Celje, Oblakova ulica, 3000 Celje, Slovenia; 3 Clinical Department of Intensive Internal Medicine, University Medical Centre Ljubljana, Zaloška ulica, 1000 Ljubljana, Slovenia

## Abstract

**Introduction:**

Our aim was to determine long-term survival and quality of life of patients admitted to a surgical intensive care unit (ICU) because of sepsis or trauma.

**Methods:**

This was an observational study conducted in an 11-bed, closed surgical ICU at a 860-bed teaching general hospital over a 1-year period (January 2003 to December 2003). Patients were divided into two groups according to admission diagnoses: group 1 included patients with sepsis; and group 2 included patients with trauma (polytrauma, multiple trauma, head injury, or spinal injury). Quality of life was assessed after 2 years following ICU admission using the EuroQol 5D questionnaire.

**Results:**

A total of 164 patients (98 trauma patients and 66 patients with sepsis) were included in the study. Trauma patients were younger than patients with sepsis (53 ± 21 years versus 64 ± 13 years; *P *≤ 0.001). There was no significant difference between groups in Acute Physiology and Chronic Health Evaluation II score or length of stay in the surgical SICU. Trauma patients stayed longer on the general ward (35 ± 44 days versus 17 ± 24 days; *P *< 001). Surgical ICU survival, in-hospital survival, and post-hospital and cumulative 2-year survival were lower in the sepsis group than in the trauma group (surgical ICU survival: 60% versus 74%; in-hospital survival: 42% versus 62%; post-hospital survival: 78% versus 92%; cumulative 2-year survival: 33% versus 57%; *P *< 0.05). There was no significant difference in quality of life in all five dimensions of the EuroQol 5D between groups: 60% of patients had signs of depression, almost 60% had problems in usual activities and 56% had pain.

**Conclusion:**

Patients with sepsis treated in a surgical ICU have higher short-term and long-term mortality than do trauma patients. However, quality of life is reduced to the same level in both groups.

## Introduction

Intensive care units (ICUs) serve patients with acute physiological derangement and organ failure but who have potentially reversible disease. They also provide a service for those who can benefit from more comprehensive observation and complex treatment than is available on standard hospital wards [1-3].

Critical illness is associated with a wide array of serious and troublesome long-term sequelae that may interfere with achieving optimal patient-centred outcomes. Although traditional short-term outcomes, such as hospital mortality, remain extremely important, they are unlikely to be adequate surrogates for subsequent patient-centred outcomes. As such, it is important to focus specifically on how critical illness and intensive care can affect long-term health and well being [4].

Critical care patients differ with respect to the reason for their admission to the ICU, and these differences are likely to result in different expectations regarding their health. Therefore, when measuring outcomes in intensive care patients, we should take into account the possibility that outcomes will vary by admission diagnostic category [5]. The aim of the present study was to compare the long-term survival and quality of life of patients treated in a surgical ICU because of sepsis or trauma.

## Materials and methods

In an observational study we included patients admitted to the surgical ICU over a 1-year period (January 2003 to December 2003). The surgical ICU at our 860-bed teaching general hospital is an adult, 11-bed, closed unit. Permission to perform the study was granted by the national ethics committee.

Patients were divided into two groups according to admission diagnoses: group 1 included patients with sepsis (severe sepsis and septic shock) and group 2 included those with trauma (polytrauma, multiple trauma, head injury, or spinal injury). Sepsis was defined as clinical signs suggesting the presence of systemic inflammatory response syndrome together with definite evidence of infection. Systemic inflammatory response syndrome was defined as any two of the following [6-8]: temperature >38°C or <36°C; heart rate >90 beats/min; respiratory rate >20 breaths/min or arterial carbon dioxide tension <32 mmHg; and white blood cell count >12,000 cells/mm^3 ^or <4000 cells/mm^3^, or with >10% immature (band) forms. Sepsis was considered severe when it was associated with organ dysfunction, hypoperfusion, or hypotension [7]. Septic shock was defined as sepsis with hypotension despite adequate fluid resuscitation, combined with perfusion abnormalities that may include lactic acidosis, oliguria, or an acute alteration in mental status [7]. Polytrauma was defined as injury to several physical regions or organ systems, where at least one injury or a combination of several injuries was life threatening, with the severity of injury being 16 or greater on the Injury Severity Score [9]. Multiple trauma was defined as physical insults or injuries occurring simultaneously in several parts of the body, and it was differentiated from polytrauma because multiple trauma was not life threatening [9,10].

Patient demographic characteristics, length of stay (LOS) in the surgical ICU, severity of illness (risk for hospital mortality, as indicated by Acute Physiology and Chronic Health Evaluation [APACHE] II score) and LOS in the general ward were all recorded, as were surgical ICU and hospital outcome. When patients were readmitted to the surgical ICU, data from their last admission were used. The hospital patient's registry was used to obtain information on patients who had died in the hospital. Survival data for patients 2 years after ICU treatment were collected from the Central Register of Inhabitants for the Republic of Slovenia.

Quality of life was assesed after 2 years following ICU admission for surviving patients using EuroQol 5D questionnaire and a telephone interview. EuroQoL 5D [11-13] is a generic health status measure that consists of three parts: a descriptive system, a visual-analogue scale, and an EuroQol 5D Index. The descriptive system measures health in five dimensions: mobility, self-care, usual activities, pain/discomfort and anxiety/depression. Patients mark one of three levels of severity (1 = no problems, 2 = some/moderate problems and 3 = severe/extreme problems) in each dimension. Combinations of these categories define a total of 243 different health states. The EuroQol 5D Index [14], based on the five dimensions and ranging from -0.11 ('worse than death') to 1 ('perfect health'), was also calculated. In the present study we used only the descriptive system and the EuroQol 5D Index.

Patients or family members of patients who were discharged alive were contacted by phone to determine their willingness to participate in the study and were asked to complete the questionnaire. In the case of severe disability of patients, we used proxy assessment of health-related quality of life (HR-QOL) [4,15].

### Statistical analysis

The characteristics of the study population were assessed using the two-way Student's *t*-test for continuous and nonparametric Mann-Whitney U-test for noncontinuous variables. Results are presented as mean ± standard deviation (range).

The survival of patients is displayed graphically using Kaplan-Meier curves, and any differences between curves were explored using log-rank or Tarone-Ware tests. For correction of age between two groups of patients (patients with sepsis and patients with trauma), Cox survival analysis was used.

For assessing differences in baseline characteristics between responders and nonresponders to the EuroQol 5D questionnaire, we used independent samples *t*-test. Analysis of variance was used to test for mean difference in EuroQol 5D profile between patients with sepsis and patients with trauma. *P *< 0.05 was considered statistically significant.

A statistical computer program (SPSS, version 13; SPSS Inc., Chicago, IL, USA) was employed to conduct data analysis.

## Results

Between January 2003 and December 2003, a total of 164 patients were included in the study. Sixty-six of the patients were admitted because of sepsis (severe sepsis and septic shock), and 98 of the patients were admitted after trauma (polytrauma, multiple trauma, head injury, or spinal injury). Gastrointestinal surgery (complications of gastrointestinal surgery, emergency gastrointestinal surgery and pancreatitis) accounted for 76% of all patients in the sepsis group.

The demographic characteristics of the patients, stratified by admission diagnosis, are shown in Table 1. The two groups of patients differed in some respects. Trauma patients were younger than patients with sepsis (53 ± 21 years versus 64 ± 13 years; *P *< 0.001). There were significantly more men in the trauma than in the sepsis group (71% versus 49%; *P *= 0.006). There was no significant difference between groups in APACHE II score or surgical ICU LOS. Trauma patients stayed longer on the general ward (35 ± 44 days versus 17 ± 24 days; *P *< 0.001).

**Figure 1:**
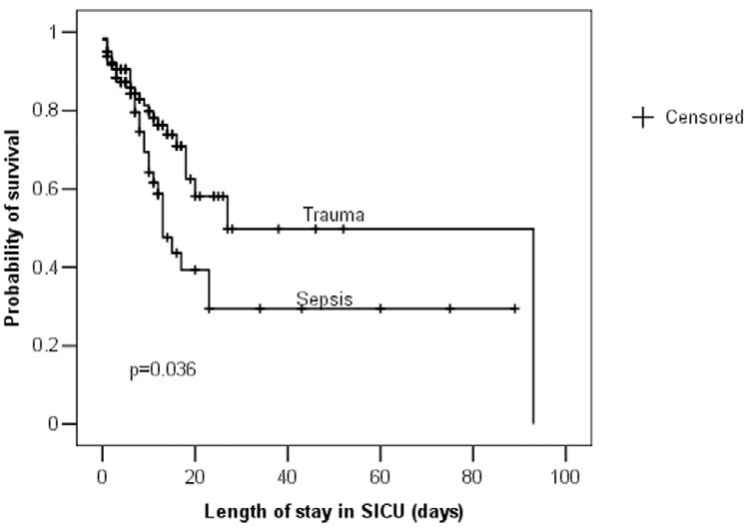
Surgical ICU mortality. The Kaplan-Meier survival curve shows that patients with sepsis had higher surgical ICU mortality than did patients with trauma. ICU, intensive care unit.

**Figure 2:**
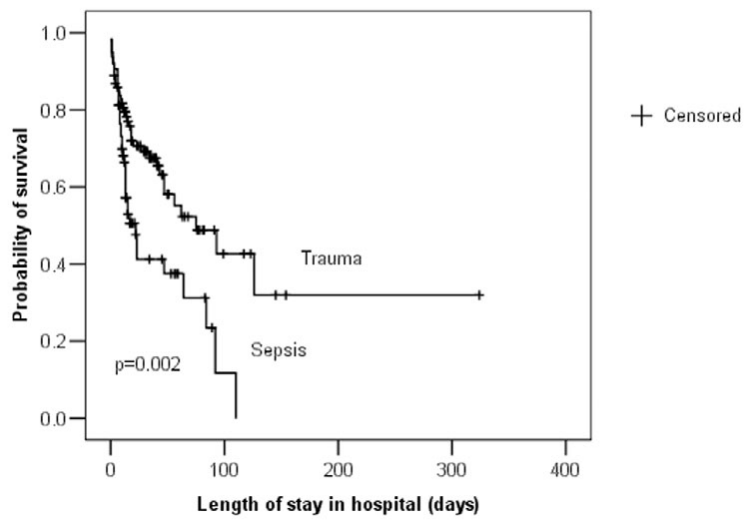
In-hospital mortality. The Kaplan-Meier survival curve shows that patients with sepsis had higher in-hospital mortality than did patients with trauma.

**Figure 3:**
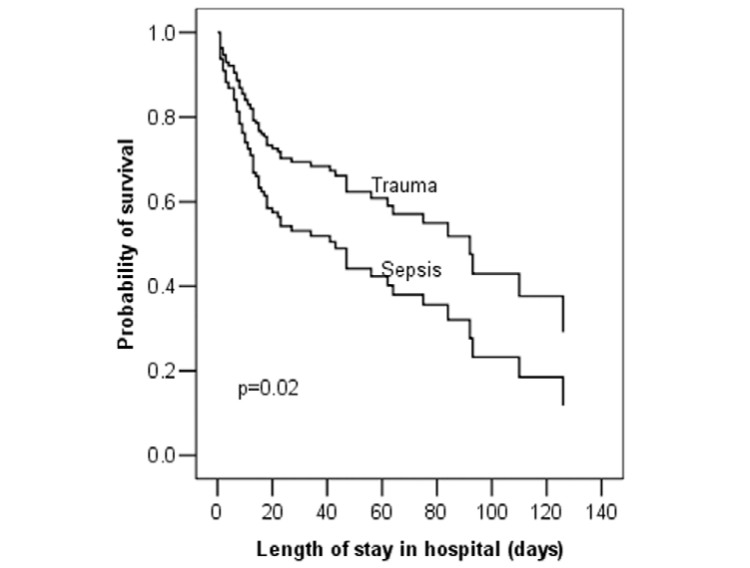
In-hospital mortality, controlling for age. The Cox survival analysis shows that patients with sepsis had higher in-hospital mortality than did trauma patients, after controlling for age.

Mortality analysis was separated into short-term mortality (surgical ICU and in-hospital mortality) and long-term mortality (post-hospital mortality). Surgical ICU mortality in the sepsis group was higher than that in the trauma group (40% versus 26%; *P *= 0.036; Figure 1). In-hospital mortality of patients with sepsis was also higher than that of patients with trauma (58% versus 38%; *P *= 0.002; Figure 2). Patients with sepsis were older than trauma patients (Table 1). To evaluate the independent effects of age and admission diagnosis (sepsis or trauma) on mortality, we conducted a Cox survival analysis. After controlling for age, we found that admission diagnosis had a significant effect on in-hospital survival (hazard ratio 1.73; *P *= 0.02; Figure 3).

**Figure 4:**
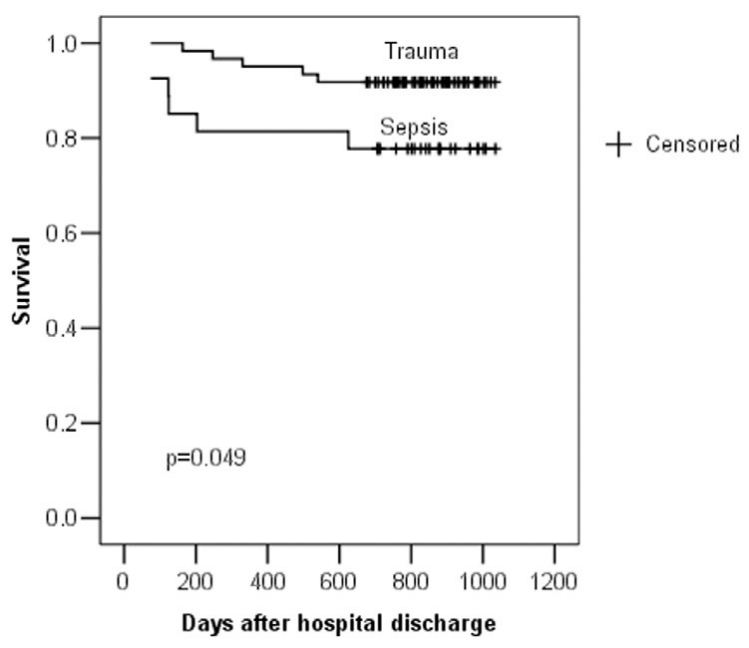
Post-hospital mortality. The Kaplan-Meier survival curve shows that patients with sepsis had higher post-hospital mortality than did patients with trauma.

**Figure 5:**
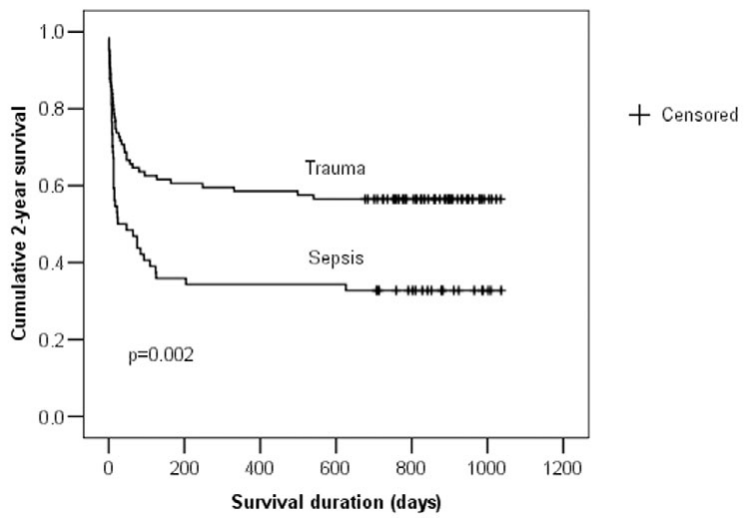
Cumulative 2-year mortality. The Kaplan-Meier survival curve shows that patients with sepsis had higher cumulative 2-year mortality than did patients with trauma.

The long-term outcomes of patients with sepsis and trauma were also significantly different. Not only did the patients with sepsis suffer a higher surgical ICU and in-hospital mortality, but these patients also had poorer long-term outcomes. Figure 4 illustrates the survival of patients after hospital discharge. Post-hospital mortality of patients with sepsis was higher than that of patients with trauma (22% versus 8%; *P *= 0.049; Figure 4). Finally, cumulative 2-year mortality was higher in the sepsis group than in the trauma group (67% versus 43%; *P *= 0.002; Figure 5).

Of the 89 patients who were discharged from hospital, 11 (12%) died during the 2-year follow-up period. Quality of life was assessed in 39 patients (50%) of the 78 surviving patients 2 years after ICU admission. In all, 48% (10/21) patients with sepsis and 51% (29/57) patients with trauma participated in the HR-QOL assessment. In addition, baseline characteristics of responders and nonresponders to EuroQol 5D are summarized in Table 2. There was no significant difference in age, sex, LOS in the surgical ICU and hospital between the two groups (Table 2). Only APACHE II score was higher in nonresponders (13.7 ± 5.9 versus 11.0 ± 4.3; *P *< 0.02; Table 2).

**Figure 6:**
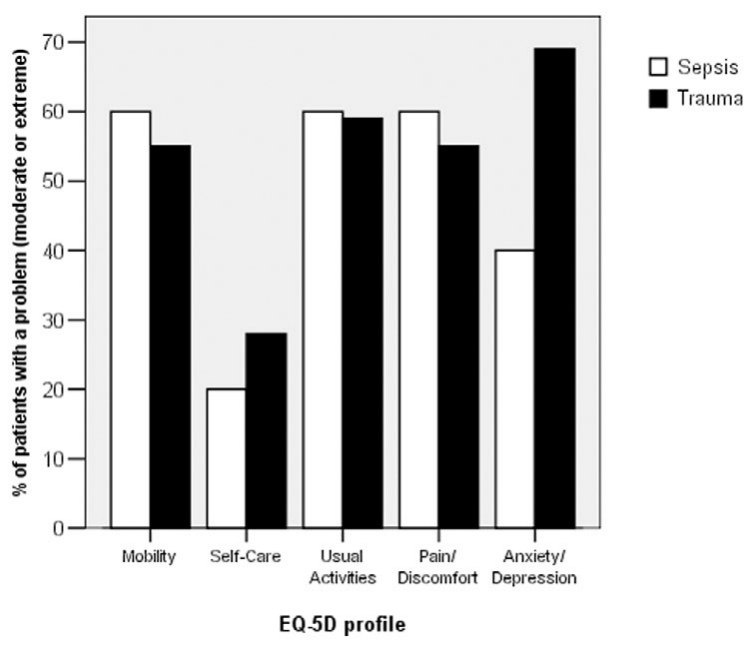
EuroQol 5D profile after 2 years. The EuroQol 5D profile is dichotomized into 'no problems' and 'moderate/extreme problems'. After 2 years there was no significant difference between the sepsis group and the trauma group.

Mean (± standard deviation) EuroQol 5D Index was 0.72 ± 0.24 and did not differ between patients with sepsis and those with trauma. One trauma patient described her functional status as being worse than death (EuroQol 5D Index = -0.11). Eighty-two per cent of patients reported having a problem (moderate or extreme) in at least one dimension of EuroQol 5D. There was no significant difference in quality of life in all five dimensions of EuroQol 5D between groups (Figure 6). Almost 60% of patients reported having problems in usual activities, 56% had pain and 56% had mobility problems; in contrast, most of patients (74%) reported having no problems in self-care. Depression and anxiety were more often detected in trauma patients, but the difference relative to the sepsis group was not significant (*P *= 0.1).

## Discussion

Two main findings of the present study may be identified. First, patients with sepsis treated in the surgical ICU have higher short-term (surgical ICU and in-hospital) and long-term (post-hospital) mortality than do trauma patients. Not surprisingly, cumulative 2-year mortality was higher in the sepsis group than in the trauma group. Severe sepsis is a life-threatening complication of infection. Becaue of the associated organ failure, treatment in the ICU is usually necessary. Several studies have shown that sepsis is the leading cause of mortality for patients admitted to ICUs [16-19]. The observation that sepsis reduces long-term survival when compared with trauma is also consistent with observations from other studies.

Davidson and colleagues [20] compared two specific groups of patients with adult respiratory distress syndrome (ARDS) and demonstrated that sepsis-ARDS patients had a sixfold higher late (post-hospital) mortality rate than did trauma-ARDS patients.

There were some differences in baseline characteristics between the two groups of patients in our study. Patients with sepsis were older than the trauma patients. Sepsis is highly correlated with age and comorbidities, and it may be difficult to separate their independent effects on mortality [21]. Davidson and coworkers [20] also argued that critical care outcome could only be measured in patients who were selected for admission to the ICU, and therefore only reflected the outcome of a subset of elderly patients. As shown in Figure 3, even after controlling for age, sepsis as the admission diagnosis had a significant effect on in-hospital mortality in our study.

The second main finding of our study is that, although patients with sepsis had higher long-term mortality and were older then the trauma patients, there was no significant difference in quality of life after 2 years following intensive treatment. Patients with trauma even had a tendency toward greater anxiety and depression compared with patients who had sepsis. Therefore, quality of life was reduced to the same level in both groups, and 82% of patients reported a problem (moderate or extreme) in at least one dimension of EuroQol 5D, but most patients (74%) reported no problems in self-care.

Critical care patients differ with respect to the cause of their admission to the ICU. We may presume that most patients with trauma were previously healthy individuals who suffered a severe injury and were admitted to the surgical ICU as a result of acute, life-threatening insults. Badia and coworkers [15] reported that trauma patients experienced considerable worsening in their quality of life, because the price of their survival was a deterioration in their HR-QOL as a result of the injuries sustained. On the other hand, patients with sepsis are usually more chronically ill and consequently have diminished HR-QOL, and are admitted to the surgical ICU because of exacerbation of pre-existing chronic pathologies or for surgical interventions aimed at improving their HR-QOL. We must emphasize that our study was conducted in a surgical ICU, and the patients with sepsis (76%) were mostly admitted after major abdominal surgery and had abdominal sepsis. Haraldsen and Andersson [22] retrospectively evaluated long-term outcomes in patients treated in a surgical ICU for abdominal sepsis. They reported that most patients who had survived after treatment of abdominal sepsis in the surgical ICU regained good health and restored functional status.

Granja and coworkers [23] also compared the HR-QOL of survivors from severe sepsis and septic shock with HR-QOL in others who had survived critical illness not involving sepsis using EuroQol 5D. They reported that survivors from severe sepsis and septic shock had similar HR-QOL to that of survivors from critical illness admitted without sepsis. In contrast to those studies, Davidson and colleagues [24] compared HR-QOL in ARDS survivors who had sepsis as their primary risk factor for ARDS with HR-QOL in ARDS survivors with trauma as their primary risk factor for ARDS. They concluded that sepsis-ARDS patients had worse HR-QOL than did trauma-ARDS patients.

The present study has limitations. No patients were lost to follow up for the assessment of survival, but 50% were lost to follow up for the assessment of quality of life at 2 years. Why did we lose so many patients? In our assessment of quality of life we used telephone interview, and for the most part those patients who were lost to follow up had no telephone or had changed their telephone number so we could not contact them. In addition, patients or relatives who refused to cooperate in the study were also lost to follow up. We believe that although we lost so many patients, our population of patients who underwent HR-QOL assessment was representative; in our comparison of responders with nonresponders to the EuroQol 5D questionnaire, we identified no differences in baseline demographics (age or sex) or in surgical ICU or hospital LOS between the two groups of patients.

A further potential limitation of this study was its inability to assess baseline HR-QOL of patients before surgical ICU admission. We assessed quality of life in surviving patients 2 years after ICU admission; this is quite a long period, and recollection of baseline HR-QOL before surgical ICU admission after such an interval may not be accurate. Grady [25] suggested that researchers should be cautious, emphasizing that recall of status at an earlier time point could be influenced by the current situation.

EuroQol 5D was the instrument of choice because it is a simple and short questionnaire that is easily understood and answered by the patients. It is a generic HR-QOL instrument that, apart from permitting estimation of an overall quality of life index, specifically measures a range of physical and nonphysical dimensions [5,11-13]. Its usefulness and construct validity have been tested in patients in several studies [15,26-32]. In addition, it is an appropriate method for measuring HR-QOL in critically ill patients [4]. Telephone assessment of EuroQol 5D has the advantage of avoiding the need to recall patients to the hospital; telephone assessment of EuroQol 5D was also validated in several studies [4,30,31]. Badia and coworkers [15] also reported that proxy responses could be reliably used with the EuroQol 5D when measuring change in HR-QOL.

## Conclusion

In summary, two main conclusions may be drawn from the study. Patients with sepsis treated in the surgical ICU have higher in-hospital and post-hospital mortality than do trauma patients, but quality of life is reduced to the same level in both groups. This means that survival and quality of life after critical illness are independent; when evaluating outcome after intensive care, it is necessary to incorporate assessments of both quality of life and survival.

## Key messages

• We studied long-term outcomes in patients with the two most frequent admission diagnoses admitted to the surgical ICU: sepsis and trauma.

• Patients with sepsis had higher in-hospital and post-hospital mortality than did trauma patients.

• Quality of life, as measured using the EuroQol 5D questionnaire, was reduced to the same level in both groups, and 82% of patients reported a problem in at least one dimension of the EuroQol 5D.

## Abbreviations

APACHE = Acute Physiologic and Chronic Health Evaluation; ARDS = adult respiratory distress syndrome; HR-QOL = health-related quality of life; ICU = intensive care unit; LOS = length of stay.

## Competing interests

The authors declare that they have no competing interests.

## Authors' contributions

HKJ was involved in the design of the study; in the acquisition, analysis and interpretation of data; and drafted the manuscript. KJ helped to draft the manuscript. MP participated in the design of the study, helped to perform statistical analysis and revised the manuscript critically. All authors read and approved the final manuscript.

## Figures and Tables

**Table 1:** The demographic details of the patients categorized by surgical ICU admission diagnostic category

Admission diagnostic category	Number of patients	Age (years)	Male sex (*n *[%])	APACHE II score	Days in the surgical ICU	Days in the general ward
Sepsis	66	64.4 ± 13.5*	32 (49)**	15.5 ± 6.4	11.8 ± 16.1	17.3 ± 23.5***
Trauma	98	53.2 ± 21.5	70 (71)	14.3 ± 6.6	11.9 ± 12.5	35.0 ± 44.2

Data are expressed as mean ± standard deviation, unless otherwise indicated. *P < 0.001, **P = 0.006 and ***P = 0.001, versus trauma. APACHE, Acute Physiology and Chronic Health Evaluation; ICU, intensive care unit.

**Table 2:** Comparison of responders with nonresponders to the EuroQol 5D questionnaire

EuroQol 5D questionnaire	Number of patients (%)	Admission diagnosis	Age (years)	Male sex (*n *[%])	APACHE II score	Days in surgical ICU	Days in hospital
Responders	39 (50)	Trauma: 29 Sepsis: 10	45.0 ± 19.8	25 (64)	11.0 ± 4.3*	11.4 ± 14.4	40.0 ± 52.8
Nonresponders	39 (50)	Trauma: 28 Sepsis: 11	52.4 ± 18.3	26 (69)	13.7 ± 5.9	10.1 ± 8.6	37.0 ± 35.4

Data are shown as mean ± standard deviation, unless otherwise indicated. APACHE, Acute Physiology and Chronic Health Evaluation; ICU, intensive care unit. *P = 0.02 versus nonresponders.
